# Fibroblast Growth Factor 21 in Gestational Diabetes Mellitus and Type 2 Diabetes Mellitus

**DOI:** 10.1155/2023/4024877

**Published:** 2023-10-14

**Authors:** Katarzyna Gawlik, Tomasz Milewicz, Dorota Pawlica-Gosiewska, Iwona Trznadel-Morawska, Bogdan Solnica

**Affiliations:** ^1^Department of Clinical Biochemistry, Jagiellonian University Medical College, Krakow, Poland; ^2^Department of Gynecology and Endocrinology, University Hospital, Krakow, Poland; ^3^Department of Metabolic Diseases, University Hospital, Krakow, Poland

## Abstract

**Objective:**

Women who develop GDM present a metabolic condition similar to that found in type 2 diabetes, characterized by impaired insulin response. Due to similar pathophysiologic mechanisms found between type 2DM and GDM, there is a great interest in finding markers that will lead to the understanding of a possible common origin to both diseases. The aim of this study was to determine serum FGF21 levels in 2DM and GDM and its correlation with selected metabolic parameters.

**Method:**

The study included 54 2DM patients and 52 nondiabetic individuals (control group 1) as well as 74 GDM women and 32 healthy pregnant controls (control group 2). Serum FGF21 was determined by enzyme-linked immunosorbent assay (ELISA), in all groups, and correlated with biochemical parameters of glucose metabolism and insulin resistance (HbA1c, HOMA index, TG, and HDL cholesterol).

**Results:**

FGF21 concentration was significantly higher in 2DM as compared with control group 1 (*p* < 0.01). In the 2DM group, FGF21 was positively correlated with HOMA index (*p* = 0.022, *R* = 0.398). In the GDM group, the positive relationships with FGF21 were observed with glucose (*p* = 0.020, *R* = 0.264) and TG (*p* = 0.013, *R* = 0.283) while HDL-C levels were correlated negatively (*p* = 0.004, *R* = −0.326).

**Conclusion:**

Serum FGF21 levels were significantly higher in 2DM patients than those without diabetes. Moreover, serum FGF21 levels were associated with selected metabolic parameters, suggesting that it may play acrolein glucose and lipid metabolism.

## 1. Introduction

Type 2 diabetes is the world's most common type of diabetes. It is caused by chronic high glucose levels in the blood as a result of insufficient insulin action (insulin resistance) and impaired insulin production by beta cells in the pancreas. Chronic hyperglycaemia is associated with long-term damage and failure of multiple organs—particularly the eyes, kidneys, nerves, heart, and blood vessels [1]. Because the diagnosis of type 2 diabetes is often delayed from months to years due to lack of symptoms, most of the people with newly diagnosed disease have macro- and microvascular complications [2].

The underlying mechanisms for development of GDM remain unclear, but the pathophysiology of GDM and 2DM is proposed to be similar. Both involve insulin resistance associated with inadequate insulin secretion and share the same risk factors [3].

GDM has also been shown to be a risk factor for 2DM after pregnancy. Studies have reported that GDM women are around 10 times more likely to suffer 2DM later in life, and up to half of them develop 2DM 10 years after their childbirth [4].

Recently, researchers have been putting much emphasis on the role of adipokines, secreted from adipose tissue, in the development of insulin resistance. In numerous studies, the adipokines, such as leptin and adiponectin, have been shown to affect skeletal muscle insulin sensitivity [5–7].

FGF21 is an adipokine discovered in recent years that regulates glucose and lipid metabolism. FGF21 is a hormone that enhances insulin sensitivity, lowers triglycerides, and regulates energy homeostasis [8]. FGF21 analogues have been extensively studied as pharmacological agents that promote weight loss, increase energy expenditure, and improve insulin sensitivity in animal models [9]. Higher circulating FGF21 concentrations have been associated with higher fasting plasma glucose and higher prevalence of impaired fasting glucose [10]. Animal studies also suggest that FGF21 is also a critical regulator of circulating adiponectin, a fat-derived hormone that appears to play a crucial role in protecting against insulin resistance and diabetes. FGF21 enhances both expression and secretion of adiponectin in adipocytes, increasing its serum level [11].

Metabolic role of FGF21 let researchers to speculate that this protein can play an important role in a pathogenesis of gestational and type 2 diabetes mellitus [12], and it can also be a promising therapeutic target for metabolic disease [13, 14]. However, the present state of our knowledge is too limited to allow us to clearly determine its effectiveness and beneficial effect on carbohydrate and lipid metabolism.

The aim of this study was to assess serum FGF21 concentration in patients with gestational diabetes, individuals with type 2 diabetes, and the respective control groups, as well as to determine the correlation between the studied protein and selected biochemical parameters (glucose, insulin, HbA1c, total cholesterol, HDL, and TG).

## 2. Material and Methods

The study included 54 2DM patients and 52 nondiabetic individuals (control group 1) as well as 74 GDM women in the second trimester of pregnancy and 32 healthy pregnant controls (control group 2). Individuals from the control group 1 have the same characteristics (age, gender) as 2DM patients while women from the control group 2 were matched to GDM women for maternal and gestational age. The study was designed for 80 GDM and 80 healthy pregnant women, but we have difficulties in recruiting healthy pregnant women during the study as we mainly cooperated with Department of Metabolic Diseases, where most of pregnant women were diagnosed with GDM.

Diabetes mellitus (DM) was diagnosed as the fasting plasma glucose was either ≥7 mmol/L (126 mg/dL) or if the 2 h glucose during an oral glucose tolerance test was ≥11.1 mmol/L (200 mg/dL). GDM was diagnosed with a 1-step approach in which a standard 2 h 75 g OGTT was performed at 24-28 weeks of pregnancy. High detection of at least 1 of these measured values (fasting ≥ 5.1 mmol/L, 1 h ≥ 10.0 mmol/L, and 2 h ≥ 8.5 mmol/L) was accepted as a diagnosis of GDM [15].

Type 2 diabetic patients were recruited from the Metabolic Diseases and Diabetology Clinical Department, University Hospital, Krakow. Normoglycemic individuals from control group 1 were recruited form the Department of Diagnostic, University Hospital, in Krakow. GDM and healthy pregnant women were recruited from the Diabetology Outpatient Department, University Hospital, Krakow, and the Department of Gynecological Endocrinology and Gynecology, University Hospital, in Krakow.

The exclusion criteria for all groups included the diagnosis of chronic kidney disease, stage G5 (GFR < 15 mL/min/1.73 m^2^), active inflammation, cancer, or systemic disease.

Blood specimens were drawn following an 8-12 h period of fasting. The blood glucose, total cholesterol, HDL cholesterol, FGF21, and insulin were measured in serum while HbA1c was analyzed in EDTA plasma samples.

All routine biochemistry tests were performed on the day of blood collection. Serum samples for FGF21 and insulin measurement were aliquoted and stored at -80°C until analysis.

The blood glucose and lipids were measured in the Department of Clinical Biochemistry of the Jagiellonian University Medical College using enzymatic methods and the MaxMat PL chemistry analyzer (Maxmat S.A., Montpellier, France).

Fibroblast growth factor 21 (R&D Systems, USA) and insulin (DRG Instruments, Germany) were determined in serum with commercial enzyme-linked immunosorbent assays according to the manufacturers' instructions.

Blood HbA1c measurements were routinely performed in the Department of Diagnostics, University Hospital, Krakow, Poland, on the D-10 Hemoglobin Testing System (Bio-Rad Laboratories, Hercules, CA, USA) of ion-exchange chromatography.

Body mass index (BMI) was calculated as weight divided by squared height in the study population (GDM and healthy pregnant women − weight before pregnancy). Insulin resistance was assessed by calculating HOMA-IR using the following formula: fasting glucose (mmol/L) × fasting insulin (*μ*U/L)/22.5.

The study was approved by the Bioethical Commission of Jagiellonian University (KBET/139/B/09), and written consent forms were obtained from all participants.

### 2.1. Statistical Analysis

All data were tested for normality using Shapiro-Wilk's test. Normally distributed continuous variables were expressed as mean ± SD and categorical variables as percentages. Skewed variables were expressed as median and interquartile range.

The differences between groups were investigated by the one-way analysis of variance (ANOVA) with a post hoc Tukey HSD test, for normally distributed data, and the Kruskal-Wallis test by rank and a post hoc Dunn's multiple comparison test, for nonnormally distributed data.

The strength of the linear relationship between two variables was measured by the Pearson test. Nonnormally distributed variables such as insulin, HOMA-IR index, HbA1c, BMI, and FGF21 were logarithmically transformed before analyses to approximate normal distributions. A *p* value < 0.05 was considered as statistically significant.

Statistical calculations were made in Statistica 12.0 (StatSoft Inc.).

## 3. Results

The characteristics of the study groups are presented in Table 1. The highest glucose level was observed in the type 2 diabetes group. Significant differences between 2DM and control group 1 were also observed in lipid profile.

**Figure 1:**
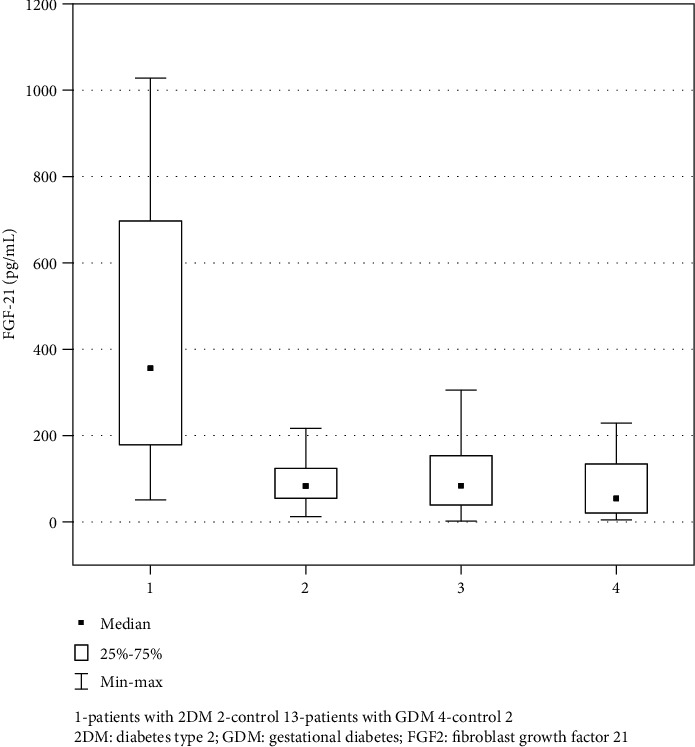
Comparison of serum FGF21 levels in patients with type 2 diabetes mellitus, gestational diabetes, and the respective control groups.

FGF21 concentration was significantly higher in 2DM as compared with control group 1. Women with GDM had higher FGF21 then non-GDM women; however, the difference was not statistically significant (Table 1 and Figure 1).

Correlation analysis (Table 2) showed a significant, positive relationship between FGF21 and HOMA-IR index in the T2DM group and with glucose concentration in the GDM group.

Analyzing the correlation between FGF21 and lipid profile, we have observed a significant, positive relationship with triglyceride levels in the GDM and non-GDM groups and also a negative with HDL cholesterol levels, in the GDM patients and control group 1.

Data are expressed as mean ± SD, median and interquartile range, or percentage of frequency (%), as appropriate.

## 4. Discussion

The aim of the study was the assessment of FGF21 concentrations in the serum of 2DM patients as well as GDM women, in comparison to controls and an attempt to evaluate the possible relationship between the studied protein and selected biochemical parameters.

The results showed that serum levels of FGF21 are significantly higher in the group of patients with 2DM compared to the control group. GDM patients had greater concentrations of FGF21 compared to control group, but the difference was not statistically significant. A study also showed a significant, positive association between circulating FGF21 levels, HOMA-IR (2DM group), and glucose (GDM group). Moreover, there was a relationship between FGF21 and adverse lipid results (TG and HDL-C) in the GDM group.

According to the results of the present research, serum FGF21 concentration was significantly higher in 2DM as compared with nondiabetic individuals (control group 1). This is consistent with numerous reports in which FGF21 levels were also observed to be higher in type 2 diabetic patients than in subjects free from diabetes [16, 17]. Additionally, some papers indicate the potential role of FGF21 as an early predictor of type 2 diabetes development [18].

Similar to the type 2 diabetic patients, FGF21 levels in the GDM group were also higher compared to healthy pregnant women. However, the differences between the groups were not statistically significant. The same results have been obtained by Stein et al. [19], but the later works revealed significantly higher levels of studied protein in GDM patients [20, 21]. The small number of participants in our control group could influenced the study results as statistically significant findings are usually harder to detect with small sample sizes.

To analyze the reasons for FGF21 elevation in both type of diabetes, the most probable explanation is a compensatory mechanism for the body, similar to hyperinsulinemia, to improve the pathological state (a.o. insulin resistance that occurs in both types of diabetes). On the other hand, the insulin resistance might cause resistance to FGF21, leading to compensatory upregulation of this antidiabetic adipocytokine [22].

Another aim of our study was to assess correlations between FGF21 levels and selected biochemical parameters. Like other authors [23, 24], we have not observed FGF21 to be correlated with insulin levels when analyzing insulin resistance markers. What is more, our results indicated that circulating levels of FGF21 increase as glucose tolerance impairs because of insulin resistance (expressed as HOMA index) in patients with T2DM, what is in agreement with the findings obtained by Bobbert et al. [25].

In the current study, insulin resistance is not significantly different between GDM and non-GDM control subjects and we failed to find a significant relation between FGF21 and insulin resistance in GDM women. This finding is in accordance with [26] but contrast to several previous studies indicating that FGF21 correlated positively with insulin resistance [27].

The present results also indicated that the serum levels of FGF21 were positively correlated with the concentration of blood glucose in GDM patients. We have not observed this relationship in 2DM, what is consistent with the study by Shafaei et al. [16], but it is not consistent with another report, where FGF21 and glucose were significantly correlated [23]. This inconsistency is probably due to the fact that the studied group was patients with newly diagnosed 2DM while our patients have been diagnosed with diabetes more than 5 years. The presence or absence of the correlation between FGF21 and glucose is not the only difference in papers. There is also different information about the direction of this relationship. Some authors report that this relationship is positive [25, 28] while others report that it is negative [29]. It can be explained by the fact that fasting blood glucose in diabetic patients has high variability, which can affect the correlation between glycemia and FGF21 [30].

In line with previous findings [19, 31, 32], we have found that serum FGF21 levels were positively correlated with TG and negatively with HDL-C in the GDM group. The paradoxical increase of serum FGF21 in GDM patients may also indicate a compensatory, protective response to adverse lipid profile as this protein is closely related to lipid metabolism [33].

There were a few limitations in our study. The first one was lack of information about treatment and/or medication in the 2DM group which could have influenced the obtained results. The second problem was a different size of studied patient groups, especially small number of pregnant women free from diabetes. This small sample size could have caused difficulties in finding significant relationships from the data. For this reason, our findings require further confirmation.

On the basis of results obtained from our study, it can be concluded that increased FGF21 concentration observed in diabetes may result from a compensatory reaction to impaired insulin sensitivity or tissue resistance to this cytokine.

The relationships we have found between FGF21 and selected metabolic parameters (glucose, HOMA index, and lipid profile) can indicate that studied protein is a potent metabolic regulator. Differences in FGF21 results obtained by authors might suggests that FGF21 regulation and secretion is complex and still not being fully understood.

## Figures and Tables

**Table 1:** General and biochemical data of type 2 diabetic patients, gestational diabetic patients, and the respective control groups.

Parameter	Studied groups				*p* value (diabetes type 2 vs. control 1)	*p* value (gestational diabetes vs. control 2)
	Diabetes type 2 (*n* = 54)	Control 1 (*n* = 52)	Gestational diabetes (*n* = 74)	Control 2 (*n* = 32)		
Age (years)	58.7 ± 12.2	53.5 ± 15.0	29.7 ± 4.0	28.5 ± 2.5	—	—
Gender	Male: 30 (50.8%) Female: 29 (49.2%)	Male: 31 (56.4%) Female: 24 (43.6%)	Female: 80 (100%)	Female: 32 (100%)	—	—
Glucose (mmol/L)	6.82 ± 2.53	4.33 ± 0.75	4.42 ± 0.44	4.32 ± 0.58	*p* < 0.001	—
Insulin (*μ*IU/mL)	13.2 (9.27-19.7)	9.80 (7.36-12.6)	12.9 (8.62-18.47)	11.0 (7.64-13.38)	*p* < 0.001	—
HOMA-IR (mmol/L × *μ*U/mL)	3.73 (2.42-7.36)	1.78 (1.26-2.40)	2.63 (1.68-3.59)	2.0 (1.37-2.83)	*p* < 0.001	—
HbA1c (%, mmoL/moL)	8.10 (7.0-9.1) 65.0 (53.0-76.0)	—	5.45 (4.90-6.00) 36.1 (30.1-42.1)	—	—	—
TC (mmol/L)	4.37 ± 0.87	4.98 ± 1.22	6.73 ± 1.15	6.81 ± 1.00	*p* < 0.005	—
HDL-C (mmol/L)	0.82 ± 0.17	1.14 ± 0.23	1.78 ± 0.29	1.93 ± 0.31	*p* < 0.001	—
TG (mmol/L)	2.80 (1.86-3.73)	1.28 (0.94-1.57)	2.03 (1.60-2.59)	2.32 (1.82-2.57)	*p* < 0.001	—
FGF21 (pg/mL)	355.68 (178.3-697.1)	83.0 (54.5-124.0)	84.1 (38.6-153.7)	54.6 (20.4-134.6)	*p* < 0.001	—

HbA1c: glycated hemoglobin; TC: total cholesterol; HDL-C: high-density lipoprotein cholesterol; TG: triglycerides; FGF21: fibroblast growth factor 21.

**Table 2:** Correlations between fibroblast growth factor 21 and biochemical parameters in type 2 diabetes mellitus, control group 1, gestational diabetes, and control group 2.

FGF21 (pg/mL)				
	Studied groups			
Parameters	Diabetes type 2 (*N* = 59)	Control 1 (*N* = 55)	Gestational diabetes (*N* = 80)	Control 2 (*N* = 32)
Glucose (mmol/L)	*p* = 0.051 *R* = 0.342	*p* = 0.085 *R* = 0.235	*p* = 0.020 *R* = 0.264	*p* = 0.346 *R* = 0.172
Log insulin (*μ*IU/mL)	*p* = 0.171 *R* = 0.244	*p* = 0.784 *R* = −0.038	*p* = 0.824 *R* = 0.026	*p* = 0.412 *R* = −0.150
Log HOMA (mmol/L × *μ*U/mL)	*p* = 0.022 *R* = 0.398	*p* = 0.709 *R* = 0.052	*p* = 0.534 *R* = 0.072	*p* = 0.645 *R* = −0.085
Log HbA1c (mmoL/moL)	*p* = 0.079 *R* = 0.310	—	*p* = 0.191 *R* = 0.151	—
TC (mmol/L)	*p* = 0.577 *R* = 0.101	*p* = 0.747 *R* = 0.045	*p* = 0.145 *R* = 0.209	*p* = 0.579 *R* = −0.102
HDL-C (mmol/L)	*p* = 0.378 *R* = −0.158	*p* = 0.003 *R* = −0.394	*p* = 0.004 *R* = −0.326	*p* = 0.376 *R* = −0.162
Log TG (mmol/L)	*p* = 0.428 *R* = 0.143	*p* = 0.145 *R* = 0.120	*p* = 0.013 *R* = 0.283	*p* = 0.006 *R* = 0.473

FGF21: fibroblast growth factor 21; HbA1c: glycated hemoglobin; TC: total cholesterol; HDL-C: high-density lipoprotein cholesterol; TG: triglycerides.

## Data Availability

The data that support the findings of this study are available on a Jagiellonian University server (link: https://chmura.cm-uj.krakow.pl/index.php/s/Svgj8Ks7XkHV0o7). Password is available on request.

## Abbreviations

BMI: Body mass index
ELISA: Enzyme-linked immunosorbent assay
FGF21: Fibroblast growth factor
GDM: Gestational diabetes mellitus
GFR: Glomerular filtration rate
non-GDM: Nongestational diabetes pregnancy
HBA1c: Glycated hemoglobin
HDL-C: High-density lipoprotein cholesterol
HOMA-IR: Homeostatic model assessment of insulin resistance
2DM: Diabetes mellitus type 2
OGTT: Oral glucose tolerance test
TG: Triglycerides.
